# Supplementary material for: Iterative improvement in the automatic modular design of robot swarms
Source: PeerJ Comput Sci. 2020 Dec 7;6:e322. doi: 10.7717/peerj-cs.322 (PMC7924708; doi:10.7717/peerj-cs.322)
Supplement: Supplemental Information 3 [file peerj-cs-06-322-s003.zip › argos3/doc/api/standalone/a00565_source.html]

ARGoS: plugins/simulator/entities/box\_entity.h Source File


- Main Page
- Related Pages
- Namespaces
- Classes
- Files

- File List
- File Members

# plugins/simulator/entities/box\_entity.h

Go to the documentation of this file.

```
00001 
00007 #ifndef BOX_ENTITY_H
00008 #define BOX_ENTITY_H
00009 
00010 namespace argos {
00011    class CBoxEntity;
00012 }
00013 
00014 #include <argos3/core/simulator/entity/embodied_entity.h>
00015 #include <argos3/core/simulator/entity/composable_entity.h>
00016 #include <argos3/plugins/simulator/entities/led_equipped_entity.h>
00017 
00018 namespace argos {
00019 
00020    class CBoxEntity : public CComposableEntity {
00021 
00022    public:
00023 
00024       ENABLE_VTABLE();
00025 
00026       CBoxEntity();
00027 
00028       CBoxEntity(const std::string& str_id,
00029                  const CVector3& c_position,
00030                  const CQuaternion& c_orientation,
00031                  bool b_movable,
00032                  const CVector3& c_size,
00033                  Real f_mass = 1.0f);
00034 
00035       virtual void Init(TConfigurationNode& t_tree);
00036       virtual void Reset();
00037 
00038       /*
00039        * Enables the LEDs for this entity.
00040        * Adds the LED equipped entity to the given medium.
00041        * If you don't call this method, the LEDs added with
00042        * CBoxEntity::AddLED() won't be update correctly.
00043        * @param c_medium The medium to which the LEDs must be associated.
00044        * @see CBoxEntity::AddLED()
00045        */
00046       void EnableLEDs(CLEDMedium& c_medium);
00047 
00048       /*
00049        * Disables the LEDs for this entity.
00050        */
00051       void DisableLEDs();
00052 
00061       void AddLED(const CVector3& c_offset,
00062                   const CColor& c_color = CColor::BLACK);
00063 
00064       inline CEmbodiedEntity& GetEmbodiedEntity() {
00065          return *m_pcEmbodiedEntity;
00066       }
00067 
00068       inline const CEmbodiedEntity& GetEmbodiedEntity() const {
00069          return *m_pcEmbodiedEntity;
00070       }
00071 
00072       inline CLEDEquippedEntity& GetLEDEquippedEntity() {
00073          return *m_pcLEDEquippedEntity;
00074       }
00075 
00076       inline const CLEDEquippedEntity& GetLEDEquippedEntity() const {
00077          return *m_pcLEDEquippedEntity;
00078       }
00079 
00080       inline const CVector3& GetSize() const {
00081          return m_cSize;
00082       }
00083 
00084       inline void SetSize(const CVector3& c_size) {
00085          m_cSize = c_size;
00086       }
00087 
00088       inline Real GetMass() const {
00089          return m_fMass;
00090       }
00091 
00092       inline void SetMass(Real f_mass) {
00093          m_fMass = f_mass;
00094       }
00095 
00096       virtual std::string GetTypeDescription() const {
00097          return "box";
00098       }
00099 
00100    private:
00101 
00102       CEmbodiedEntity*    m_pcEmbodiedEntity;
00103       CLEDEquippedEntity* m_pcLEDEquippedEntity;
00104       CVector3            m_cSize;
00105       Real                m_fMass;
00106       CLEDMedium*         m_pcLEDMedium;
00107 
00108    };
00109 
00110 }
00111 
00112 #endif
```

---

Generated on 10 Jul 2018 for ARGoS by 
 1.6.1
